# P339: Situational analysis of patient safety and risk management in healthcare settings in Benin

**DOI:** 10.1186/2047-2994-2-S1-P339

**Published:** 2013-06-20

**Authors:** A Attolou Gbohoun, L Assogba, E Beheton, lE Adisso, AD Gazard

**Affiliations:** 1 Health Ministry of Benin, Cotonou, Benin

## Introduction

Patient safety is a priority for public health policies promoted by the WHO at the international level. It participates in the proper management strategies to strengthen national health systems, now recognized as a prerequisite for the success of any health program. According to WHO estimates, 4-16% of hospitalized patients suffer adverse effects and 75% of these can be avoided.

## Objectives

Make an inventory of patient safety from the perspective of developing the main lines of the national strategy on patient safety and risk management and better care in Benin.

## Methods

This is a cross-sectional study referred evaluative one-day patient safety, risk management and health vigilance in 39 hospitals in Benin. The hardware consists of a questionnaire whose items are divided into nine components of the strategy of the World Health Organization. The scores of items were multiplied by 20. A Quote> 60% expressed an acceptable safety and security < 40% unacceptable.

## Results

Strengths: Existence of a directorate of hospital hygiene focused on the prevention of healthcare associated infections (69.4%), the existence of regulations to prevent nosocomial infections (95.6%), the existence of a best practices guide for health professionals (74.4%), the existence of procedures on hand hygiene (71.08%).

Weaknesses: Lack of national patient safety policy (20%), lack of a social security system for health professionals against health care-related accidents (11.4%), lack of a funding mechanism for the consequences of health care-related accidents (06.2%), organization of open days on patient safety and error reporting procedures related to care (19.06%).

## Conclusion

The analysis of the patient safety situation in Benin showed that results are obtained in some domains. However, many gaps remain. More strategies need to be implemented to ensure patient safety in Benin.

## Disclosure of interest

None declared

